# A Uric Acid Polymorph Generated via Thermal Decomposition

**DOI:** 10.1021/acs.cgd.6c00610

**Published:** 2026-07-22

**Authors:** Josephine Bicknell, Shae S. London, Talaidh L. Isaacs, Amanda K. Rutledge, Lindsey K. Foote, Alyssa M. Thornton, Timothy G. Fawcett, Jennifer A. Swift

**Affiliations:** † Department of Chemistry, 8368 Georgetown University, Washington, District of Columbia 20057, United States; ‡ International Centre for Diffraction Data, Newtown Square, Pennsylvania 19073, United States

## Abstract

Uric acid is a metabolic product of purine metabolism and a normal constituent of urine. It can exist in multiple solid forms, e.g. hydrates and salts, though solution crystallization methods have to date yielded only one anhydrous polymorph (UA, Form I). Here, we report a novel anhydrate (UA, Form III), which is generated via the solid-state decomposition of ammonium urate hydrate at temperatures above 200 °C. UA Forms I and III are topologically similar, but have *b*-axes that differ by ∼0.2 Å. The distinct *a/b* ratios create features in the X-ray powder diffraction patterns that unambiguously distinguish the two forms. A structure model for Form III was developed using Rietveld refinement methods. Differences in the electron density delocalization in the two forms suggest that Form III consists of a mix of tautomers, whereas Form I consists of molecules exclusively in the triketo form. The compositions are rationalized based on DFT calculations, which predict a significant population of higher-energy tautomers is present under the thermal conditions used to prepare Form III. By contrast, Form I crystallizes from aqueous solutions where the lowest-energy tautomer has an abundance of >99%.

## Graphical abstract

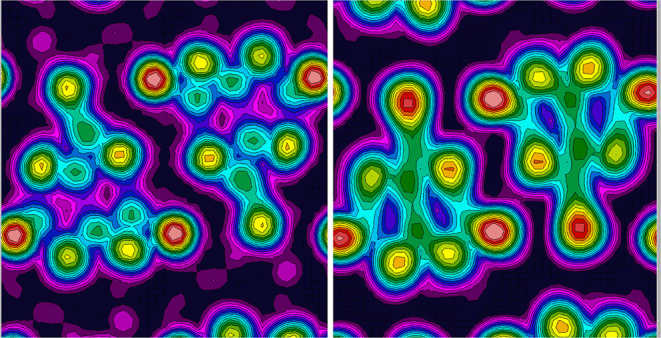

## Introduction

For over two centuries, uric acid (UA) (C_5_H_4_N_4_O_3_) has been recognized as a normal byproduct of purine metabolism.^1^ While low concentrations are eliminated naturally, elevated levels of uric acid can result in its crystallization in vivo*.* Depending on the local environment where crystallization occurs, a number of solid forms are possible. For example, anhydrous uric acid (UA) and uric aciddihydrate (UAD) are commonly identified in human kidney stones.^2,3^ Reptiles typically produce and eliminate nanocrystalline uric acid monohydrate.^4^ At higher pH, uric acid (p*K*_a_ ∼ 5.3) exists predominantly in its deprotonated form, which can crystallize as a number of urate salts. The presence of monosodium urate monohydrate crystals in synovial fluid is the classic clinical symptom of gout.^5,6^ Ammonium urate deposits are common in some breeds of domestic pets.^7–9^

Despite the many possible biogenic crystal forms, all solution growth studies to date, including hundreds in the author’s laboratory over the past 25 years, have yielded only one polymorph of UA. Its structure (refcode: URICAC) has been known since 1966 when it was first reported by Ringertz.^10^ A powder diffraction pattern for a second UA phase, Form II, was reported by Shirley and Sutor^11^ in 1968. Form II was attributed to sample changes during storage, as it was isolated from the exposed surface of a UAD stone. Dehydration of synthetic UAD^12^ also yields a diffraction pattern consistent with biogenic Form II, though the structure of Form II has remained elusive.

In previous work, we demonstrated that some of the challenges surrounding the structural chemistry of biogenic ammonium urate stem from it being a mixture of hydrate and anhydrate forms.^13^ Follow-up studies on the properties of ammonium urate hydrate (AUH) led to the serendipitous discovery that its thermal decomposition generates a novel polymorph of uric acid, UA Form III. The first new polymorph of UA in over 50 years, X-ray powder diffraction data readily distinguish Form III from Forms I and II. A structure model was developed using Rietveld refinement methods in combination with other analyses. Unlike Form I, which consists of molecules exclusively in the triketo form, we posit that Form III is composed of a mix of tautomers. The compositions are rationalized based on the different Boltzmann distributions of tautomers present under the synthetic conditions used to prepare Forms III and I.

## Experimental Section

### Materials

Uric acid (Aldrich, 99+%) and 25% ammonium hydroxide solution (Acros Organics) were used as received. All crystallization experiments used 18 MΩ UltraPure water from a Milli-Q Integral Water Purification system.

### Ammonium Urate Hydrate

Anhydrous uric acid (90 mg) was immersed in 30 mL of a room-temperature solution of 25% ammonium hydroxide for 24 h. The solid was isolated by vacuum filtration and ground by hand in a mortar and pestle. For simplicity, the authors refer to the product of this reaction as AUH, although it is known to contain smaller amounts of anhydrous ammonium urate as reported in ref 13.

UA Form III was reproducibly generated by thermal decomposition of AUH using different heat treatments at two sites. At Georgetown (GU), AUH was heated under a constant nitrogen flow in a TA Instruments Discovery DSC 25 differential scanning calorimeter at 20 °C/min to 400 °C. At the International Centre for Diffraction Data (ICDD), AUH was placed in an aluminum pan and heated in a drying oven for different time periods and at different temperatures. Samples were periodically removed from the oven for PXRD analysis.

### Uric Acid Dihydrate

UAD was crystallized from aqueous uric acid solutions (0.18 mg/mL) by slow evaporation at room temperature for 2 days as reported in ref 12. UA Form II was reproducibly generated by heating UAD in an open ceramic pan under a nitrogen flow at 5 °C/min from room temperature to 200 °C in a TA Instruments SDT Q600 instrument.

### Powder X-ray Diffraction

PXRD data were collected on hand-ground samples at two different locations. At GU, samples were prepared in Kapton capillaries with PXRD data obtained on a Bruker Apex DUO X-ray diffractometer (Cu Kα radiation, transmission mode, 50 kV, 30 mA current, 3–60°). At the ICDD, data were collected on a Bruker D2 Phaser second generation X-ray diffractometer (Cu Kα radiation, reflection mode, 300 W, 0.02° step, 1 s/step, rotated at 15 rpm, 8–70°), with a zero background holder off-cut and polished Si wafer. The range and step time were increased to 8–90° and 4 s if the data were targeted for a Rietveld refinement.

### Time-Resolved Synchrotron Powder X-ray Diffraction

Variable temperature sPXRD data on UA Form I were collected at the Advanced Photon Source on beamline 17-BM-B (λ = 0.24087 Å). Hand-ground samples were loaded into quartz capillaries (OD = 1.1 mm), maintained under a dry He flow, and continuously rocked at 10–15° throughout the experiment. Data were continuously collected from 25 to 300 °C while heating at 10 °C/min using an exposure time of 2.0 s and summation over 5 images. Image processing and integration were performed using GSAS-II^14^ and JADE Pro.^15^ Data were converted to a CuKα scale after subtracting the quartz background from the raw data.

### Rietveld Refinement

Structure refinements for Forms I and III were carried out on multiple PXRD patterns using the whole-pattern fitting module of JADE Pro. Typically, unit cells, scale factors, and an overall temperature factor were initially refined, and the peak profiles were refined in later cycles. A refinable fifth-order polynomial was applied to PXRD data collected at GU to eliminate the background signal from Kapton capillaries. A simpler third-order polynomial was used with PXRD data sets collected at the ICDD to account for the angularly dependent air scatter.

The PXRD pattern of commercial UA (attrition milled with agate media for 3 min to minimize orientation effects) refined as expected to the same unit cell as previously reported for Form I. All cell parameters were within 0.2% or less of the published value and a refined volume of 602.6 Å^3^ vs 602.9 Å^3^. The *R*_wp_ = 7.76% was limited by the quality of the experimental data (i.e., a quick scan) but was deemed sufficient to confirm the validity of the structure and its assignment as Form I UA.

Multiple data sets from thermally decomposed AUH were refined to the same Form III unit cell. An initial energy-minimized structure for UA Form III was calculated using VASP^16^ and DFT using the Form I structure (refcode: URICAC). Initial refinements matched the main features in experimental PXRD patterns, and later refinement cycles reduced the residuals and improved the molecular geometries. Final refinements on multiple data sets had an *R*_f_ between 4.78 and 6.43%. The structure reported here is from the data and determination the authors felt had the best combination of low *R*_wp_, lower errors on unit cell parameters, and a chemically reasonable structure. PDF-5+ number: 00-074-1569.

### IR Spectroscopy

Fourier-transform infrared (FT-IR) spectra were recorded on a PerkinElmer Spectrum-Two FT-IR spectrophotometer equipped with a UATR-TWO diamond ATR attachment. Each spectrum presented is an average of ten scans.

### DFT Calculations

Select tautomers of UA were built in Spartan Student V9 ^17^ and imported into Gaussian16.^18^ The tautomer geometries were optimized using the M06-2X level of theory.^19^ The single-point energies and frequencies in the gas phase and in solution were calculated using the M06-2X functional^19^ and the cc-pVQZ basis set.^20^ The continuum solvation model SMD was used to model water-solvation effects.^21^

The single-point energies were used to calculate the Boltzmann distribution at varying temperatures according to

$$P_{t}=\frac{e^{-{\Delta E}_{t}/kT}}{\sum_{i}e^{-/kT}}$$

where *P*_t_ is the relative population of a given tautomer to the entire population of all tautomers considered, Δ*E* is the difference in energy between a given tautomer and the minimum energy tautomer, *k* is the Boltzmann constant, and *T* is the temperature in Kelvin.

## Results and Discussion

Here, we show that there are at least three unique polymorphs of anhydrous UA, which can be prepared from very different routes. Representative experimental PXRD patterns for Forms I, II, and III are overlaid in Figure 1. The Form I pattern is commercial UA powder as received (Figure S2). Recrystallization of commercial material in aqueous solution at 37 °C also consistently yields UA Form I. The Form II pattern is the product formed by dehydration of UA dihydrate (UAD).^12^ The PXRD is consistent with the Form II pattern reported by Shirley and Sutor^11^ (Figure S3). The Form III pattern is the product formed by thermal decomposition of AUH.^13^ The three patterns are readily distinguishable, with the largest differences observed in the region between 2θ = 27 and 32°.

**Figure 1:**
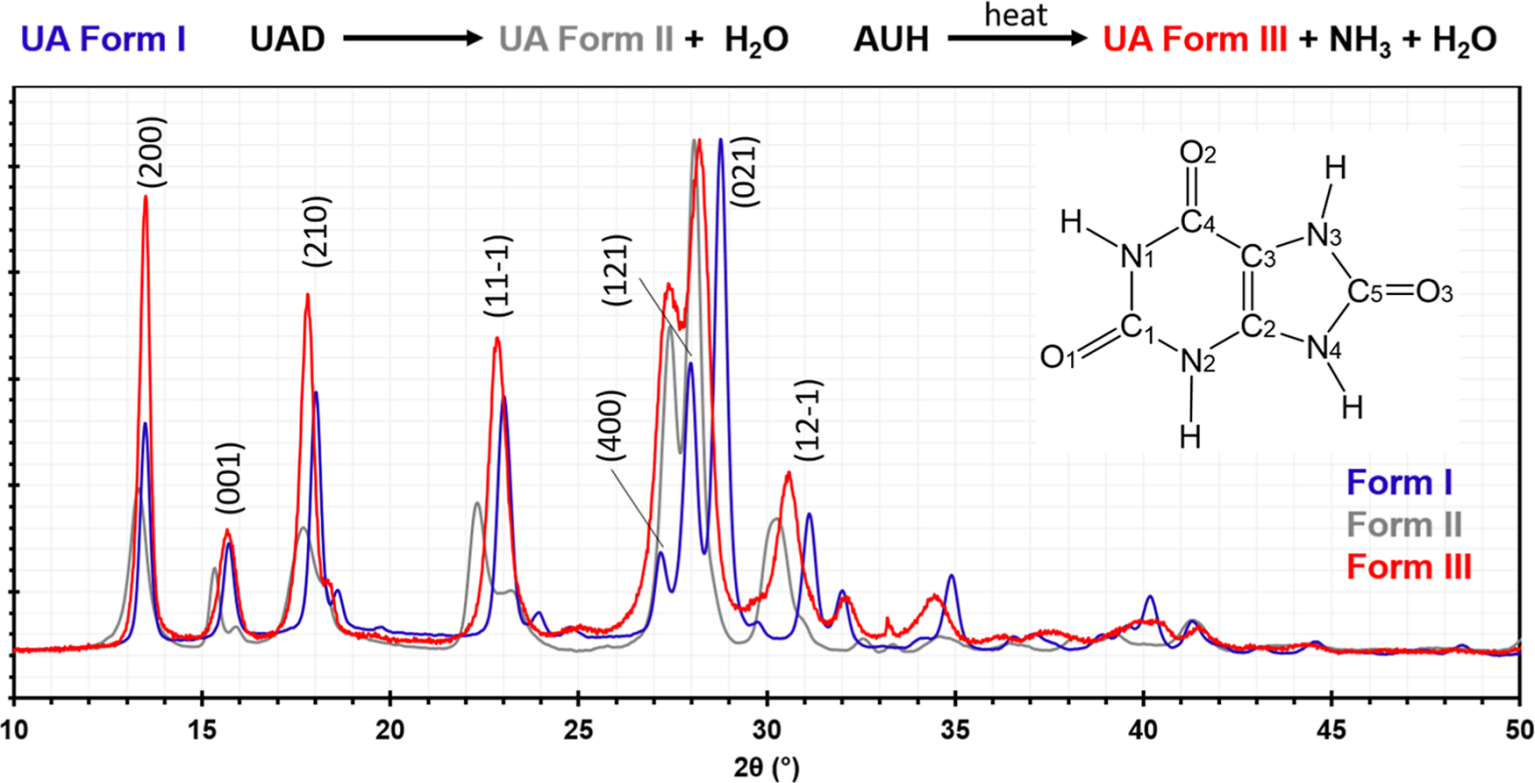
Representative experimental PXRD patterns of UA Forms I (blue), II (gray), and III (red). Form I is commercial material, Form II is dehydrated UAD, and Form III is decomposed AUH. Miller indices correspond to Form I (URICAC). For an offset view, see Figure S1.

Unlike UAD, which slowly dehydrates during storage,^12^ AUH is stable under ambient conditions and requires elevated temperatures to induce loss of ammonia and water. AUH decomposition was explored under a variety of thermal processing conditions including (a) heating for 1 h at 150, 190, 220, and 260 °C, (b) heating for up to 4 days at 150, 170, and 200 °C, and (c) heating at 20 °C/min from 25 to 400 °C (Figure S4). PXRD patterns collected after each treatment enabled assessment of the range of heating times and temperatures required to produce UA Form III.

The processing conditions in (a–c) showed a mixture of results. AUH samples held for 1 h at temperatures at or below 220 °C were unchanged. Samples heated for 1 h at 260 °C yielded mostly Form III, though peaks corresponding to unreacted AUH were still evident in the PXRD indicating that decomposition was incomplete. Longer heating times (b) or higher temperatures (c) were generally more effective. While samples heated for multiple days at or below 170 °C were unchanged, samples heated for 3 days at 200 °C showed complete transformation to UA Form III. AUH heated at 20 °C/min to 400 °C also fully transformed to UA Form III. Collectively, these results pointed to various combinations of time and temperature (≥200 °C) that were effective at decomposing AUH to UA Form III. Repeat syntheses under these conditions consistently yielded the same decomposition product.

AUH could be regenerated by immersing UA Form III in ammonium hydroxide solution, and the reconstituted AUH could be decomposed again to Form III with application of the same heat treatment (Figure S5). The IR spectra of UA Forms I, II, and III are quite similar (Figures S6 and S7), though this is not uncommon for polymorphs.^22^ Some of the peak positions in the fingerprint region do differ by a few wavenumbers, e.g. Form III has peaks at 1304 and 781 cm^–1^ while Form I has peaks at 1299 and 775 cm^–1^. Notably, the Form III spectrum shows no evidence for trapped ammonia or other potential uric acid degradation products.^23^

### Rietveld Refinement of Form III

Conventional search/match identification of the Form III powder pattern against the Powder Diffraction File (PDF-5+)^24^ with JADE PRO demonstrated a similarity to both UA Forms I and II but not a precise match. Yet, the overlap in the PXRD diffractograms, particularly at low angles, suggested that the three polymorphs share a similar topology. An attempt was made to refine the Form III structure starting from the Form I cif file (URICAC). A block refinement strategy was used^25^ where the first step refined the unit cell and scale factors, the second step refined the peak widths, and in the final step, the atomic coordinates. Since the refinement process in JADE PRO is dynamic and interactive, one can observe changes as they occur in separate steps. The initial refinement of the unit cell and peak widths caused the R-factor to drop considerably but it leveled out above 10. Most peaks were well fit, with the exception of the peaks between 2θ = 30 and 32°, which had large residuals. When all atoms were refined in the third step, the R-factor dropped below 7 but some of the bond lengths were not reasonable.

Examination of the (1 2 −1) lattice plane in the 2θ region with the large residuals showed that it slices through molecules, specifically through C(4), C(3), and N(4). Hypothesizing that these atoms were the most likely to change due to the diffraction pattern differences between Form I and Form III, the refinement strategy was altered so that these atoms were initially refined and then the surrounding atoms were refined in a three-step process. Each step noted above consisted of multiple refinement cycles until convergence, resulting in an *R*_wp_ ∼ 5.8. The experiment was repeated four more times, varying the sequence of the later steps and varying some of the peak profile and background fit parameters. In the four determinations, the final *R*_wp_ ranged from 5.44 to 6.44, there were no significant residuals, and the cell parameters were identical within the standard deviation limits. These refinements yielded molecules with reasonable bond lengths but an O2–C4–C3 angle that was unrealistically small. Adjusting the position of the C3 atom by just 0.25 Å significantly improved the molecular geometry. In the final refinement cycle, this slightly increased the C3 Biso value but did not substantially change the atomic displacement parameters for any other atom.

Refinement of PXRD patterns from multiple decomposition experiments yielded nearly identical unit cells with volumes ranging between 608 and 612 Å^3^ (Table S1). The final and best structure refinement with hydrogen atoms in assumed positions had an *R*_wp_ = 5.13 (Table 1 and Figure S8). The same atom numbering scheme as URICAC is used for comparison purposes.

**Table 1:** Unit Cell Parameters for Form I and Form III UA

	Form I	Form I	Form III
	single crystal (URICAC)	powder	powder
	*P*2_1_/*a*	*P*2_1_/*a*	*P*2_1_/*a*
*a* (Å)	14.464(3)	14.458(6)	14.31(2)
*b* (Å)	7.403(2)	7.428(6)	7.62(2)
*c* (Å)	6.208(1)	6.193(6)	6.17(2)
α (deg)	90	90	90
β (deg)	65.10(5)	64.94(2)	65.13(4)
γ (deg)	90	90	90
vol. (Å^3^)	602.94	602.57	610.4(5)
density (g/cm^3^)	1.85	1.85	1.83
a/b	1.95	1.95	1.88
*R*_f_/*R*_wp_	6.6	7.76	5.13

### Comparison of Form III and Form I

Form III is topologically similar to Form I but has a longer *b*-axis ∼0.2 Å and a shorter *a*-axis ∼0.14 Å. This gives Form III a slightly larger cell volume and hence a lower density than Form I. UA Form III and Form I structures are compared in Figure 2. Both consist of molecules assembled into 1-dimensional hydrogen-bonded ribbons formed between pyrimidine···pyrimidine (N1–H1···O1, R_2_^2^(8)) and imidazole···imidazole (N3–H3···O3, R_2_^2^(8)) groups. Parallel ribbons π-stack to create dense layers in the *bc* plane. The ribbon direction alternates between [012] and [01–2] in adjacent (*h*00) layers, creating a criss-cross pattern. In addition to the hydrogen bonding within a ribbon, each UA molecule also hydrogen bonds to four additional molecules in adjacent layers through O2···H–N4, on one side, and N4–H···O2, O3···H–N2, and N2–H···O3, on the other. This creates a strong 3-dimensional hydrogen-bonding network.

**Figure 2:**
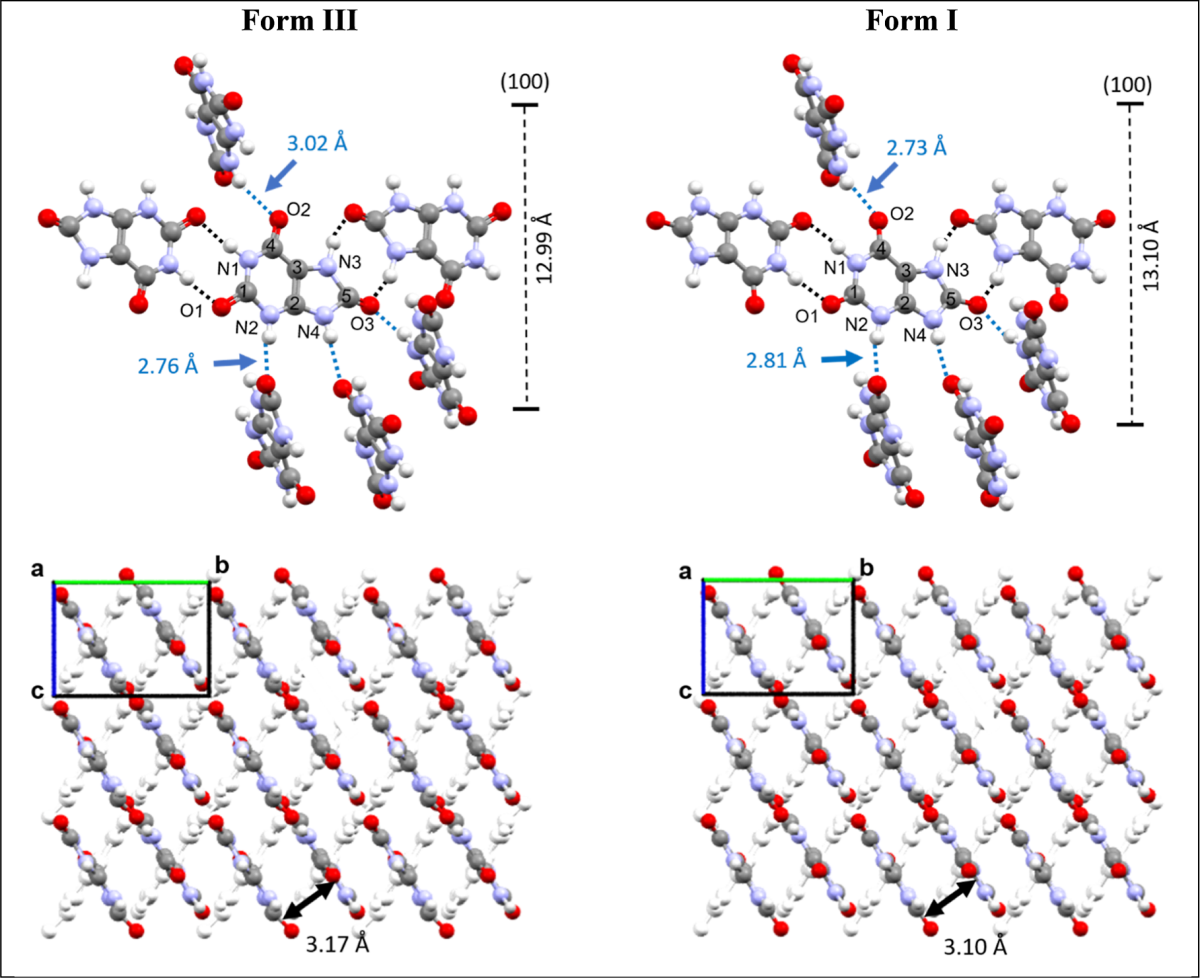
Comparison of Form III (left) and Form I (right) UA structures. The top views show the hydrogen bonding around each molecule. Hydrogen bonds within the 1-D ribbons (black) and between *bc* layers (blue) are indicated. The bottom views are of the dense *bc* plane viewed down the *a*-axis. The distance between π-stacked ribbons is indicated. The adjacent (*h*00) layer is shown in pale gray to better illustrate the criss-cross stacking.

Yet there are differences in the two forms, both within the dense *bc* planes and between them. Within each dense layer, the hydrogen bond lengths along the 1-dimensional ribbons are nearly identical (N···O: III = 2.77, 2.81 Å and I = 2.80, 2.83 Å). However, the distance between π-stacked ribbons is ∼2.3% higher in Form III than in Form I (3.17 Å vs 3.10 Å). The reduced planar density within (100) in Form III is partially offset by an increased density in the orthogonal direction. The (100) planes in Forms III and I are separated by 12.99 Å and 13.10 Å, respectively. The N2–H···O3 bond lengths in the two polymorphs are similar, but the two O2···H–N4 distances in Form III are longer by ∼0.3 Å.

It is unusual for two polymorphs to have such similar topologies yet distinct room-temperature PXRD patterns. However, attempts to convert Form III to Form I through repeat heating–cooling cycles proved ineffective. High temperature was recognized as a critical parameter for the decomposition of AUH, but why it would yield a new polymorph remained an open question. A previous study on Form I showed that thermal expansion is highest along the π-stacking direction.^26^ Extrapolating from this previous data, the 1-dimensional ribbon separation in Form I at 200 °C is comparable to the 3.17 Å repeat in Form III. However, the Form III structure model is based on room-temperature data. For Form III to retain a 3.17 Å repeat distance upon cooling, it indicated that additional factors must limit its ability to contract along that dimension. Although the causal factors cannot be determined definitively based on the available evidence, several possibilities were considered.

The molecular geometries in Forms I (colored blue) and III (colored by atom) are superimposed in Figure 3A. They show very good overlap, though the O2 position in Form III is slightly shifted. This could indicate a small rotational disorder among molecules, though attempts to include disorder in the structure model for Form III did not improve the *R*_wp_. More extensive molecular disorder in Form III seemed unlikely, given the quality of the fit between the structure model and the experimental data. Entrapment of chemical impurities in Form III was also considered. Notably, the IR spectra of Form III (Figures S6 and S7) showed no evidence for the presence of residual ammonia or other UA degradation products in the sample. To the extent that chemical impurities may be present in Form III at levels below spectroscopic detection, it seemed unlikely they would induce a consistent structure change. Previous work^27–30^ has shown that Form I can tolerate the inclusion of many different molecular additives in concentrations <1 wt % with no obvious change to the PXRD pattern.

**Figure 3:**
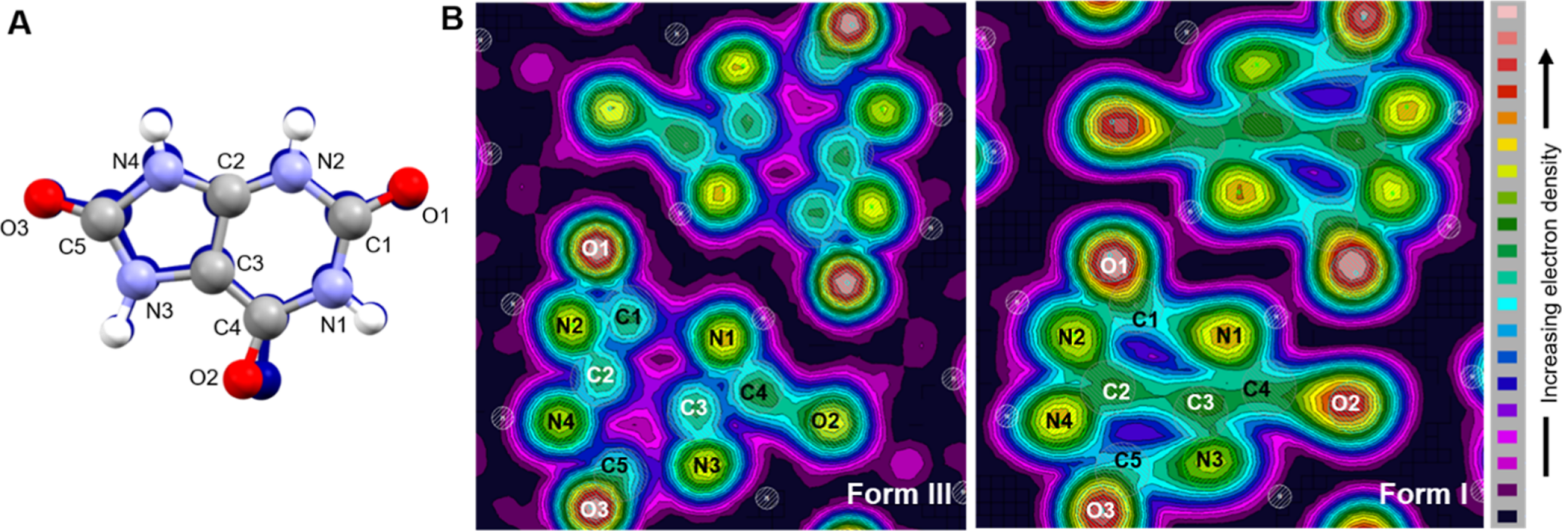
(A) Overlay of the Form I and Form III molecule geometries. Atoms in Form III are colored by atom (C = gray, N = light blue, O = red, H = white) while Form I atoms are colored blue. (B) Experimental electron density maps for Forms III and I with the positions of all heavy atoms identified. The electron density between C2 and C3 is noticably reduced in Form III relative to Form I.

Having ruled out extensive disorder and inclusion of decomposition byproducts, we considered the potential role of tautomers.^31^ With four H-bond donors and three H-bond acceptors, uric acid has many potential tautomeric forms that differ only in the H atom position. The C, N, and O positions are largely the same in all tautomers, though one expects the electron density at these atoms to be affected by the H atom positions.^32^ Electron density maps for Forms III and I appear in Figure 3B. The maps were constructed from experimental PXRD data in combination with the structure models for proper indexing of reflections. While structure models put electron density in spheres and ellipsoids, the electrons in pyrimidine ring systems are highly delocalized. The extent of delocalization emerged as a real difference between the two forms. Specifically, the electron density between the bridgehead atoms (C2 and C3) in Form III is significantly lower than that in Form I. Isosurfaces with 50, 34, and 18% rendering provide another way to visualize the differences in electron delocalization (Figure S10).

UA molecules in Form I (and UAD) are in the triketo (also known as triamido) form shown in Figures 1, 2, and 3a. Although we had originally assumed molecules in Form III were also in the triketo form, the difference in the electron density maps was a strong indication that Form III could be comprised of a mixture of tautomers. The reasonableness of that hypothesis would depend on whether a sufficient population of higher-energy tautomers exists under the synthetic conditions required to make Form III.

### Tautomer Populations

Many previous authors have calculated the relative energies of uric acid tautomers at varying levels of theory.^32–35^ All identify the triketo form as the lowest in energy in both the gas phase and aqueous solution. However, Form III is prepared under elevated temperature conditions via solid-state decomposition where urate is protonated, ammonia and water are expelled, and a new lattice is generated. Under these synthetic conditions, the relative tautomer populations may not be the same.

We focused our computational efforts on the seven most likely tautomers shown in Figure 4a. The N#_O# naming convention is derived from the atom labels in URICAC, to specify the original and new position of the H atom (in red) relative to the triketo minima. N2_O1 and N4_O3 were examined because prior studies identified them as the next lowest-energy gas-phase forms. N2_C3 and N4_C3 were considered based on previous semiempirical calculations^35^ that identified them as relevant and because they could potentially rationalize the slightly larger Biso value for C3 in the Form III model refinement. The final two, N2_O2 and N4_O2, were considered for topochemical reasons, given their proximity in adjacent (*h*00) layers. These forms would also involve a hydrogen transfer from the most acidic atoms (N2–H and N4–H) to the most basic atom (O2).^34^

**Figure 4:**
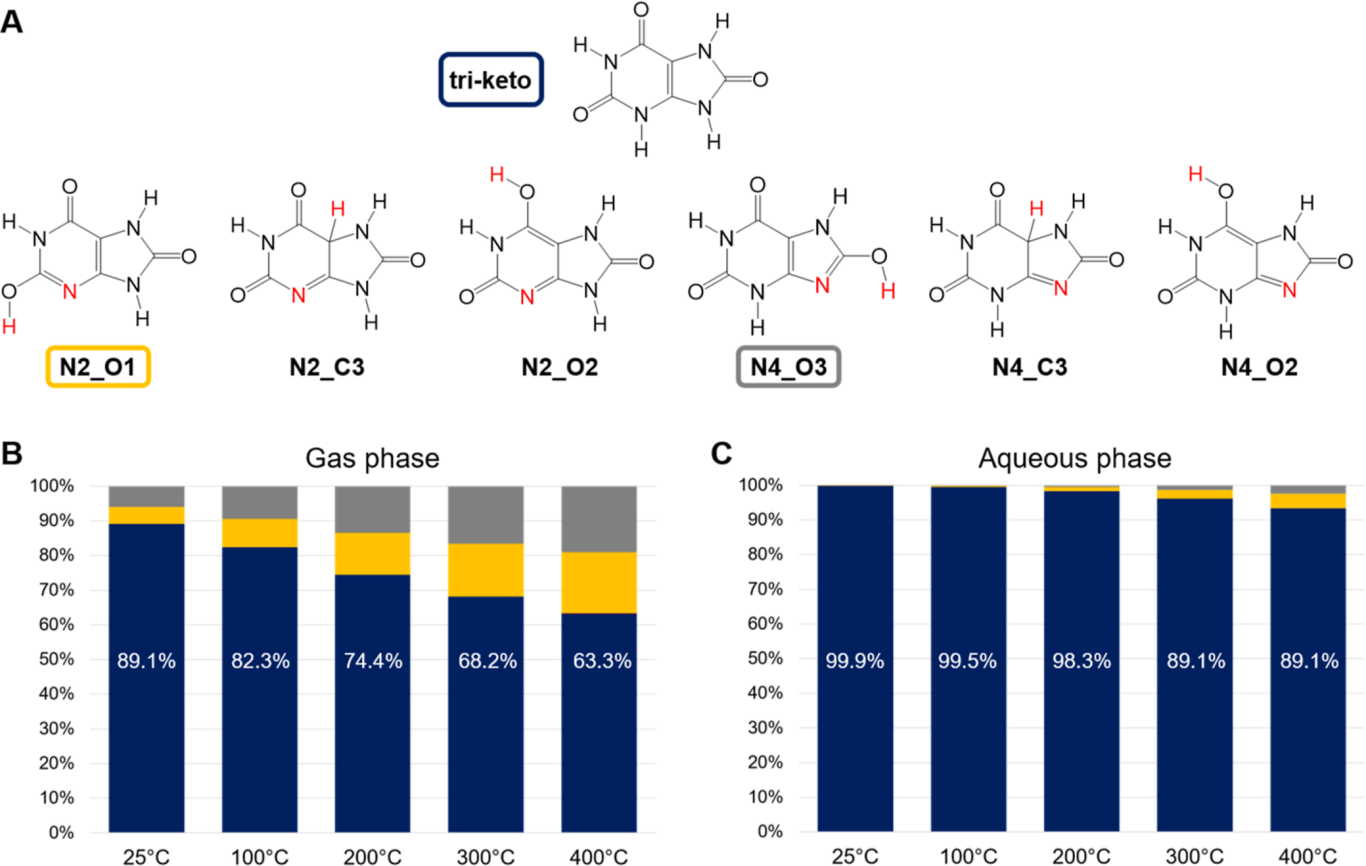
(a) Select tautomers of uric acid. (b,c) Boltzmann distribution of tautomers based on single-point energies calculated at the MO6-2X/cc-pVQZ level in the gas phase and in aqueous solution (SMD model) at temperatures between 25 and 400 °C. Triketo (navy), N2_01 (yellow), N4_03 (gray) Tautomers N2_C3, N4_C3, N2_O2, and N4_O2 each had an abundance of ≤0.1% under all conditions.

Energies of the seven tautomers were calculated in both the gas phase and aqueous phase using the M06-2X functional and cc-pVQZ basis set (Table S2). The basis set was selected for its high level of accuracy and precision^36^ since small energy differences greatly influence the relative populations. In the gas phase, the triketo tautomer was the lowest in energy, as expected. Energies for N4_O3 and N2_O1 were just 1.61 and 1.71 kcal/mol higher, respectively. The other four tautomers had much higher energies, 9.65–17.14 kcal/mol above the minima. When water solvation effects were included (SMD model), each of the six additional tautomers considered was 4.14–11.21 kcal/mol higher than the triketo form.

The single-point energies were then used to estimate the Boltzmann distribution at temperatures ranging from 25 to 400 °C (Figure 4b and Table S3). The triketo tautomer is always most abundant, though in the gas phase significant amounts of N2_O1 and N4_O3 are also present. These two minor tautomers make up an increasing fraction as the temperature increases from 25 °C (10.9%) to 200 °C (25.6%) to 400 °C (36.7%). Given the abundance of higher-energy tautomers at the temperatures needed to synthesize Form III, it seems feasible if not likely that some fraction would be kinetically trapped in the lattice. An isolated trapped N2_O1 tautomer (or any other chemical impurity) can be accommodated by local relaxation, with little or no measurable change in unit cell dimensions. However, a sufficiently high concentration of such inclusions (e.g., solid solution) would be expected to alter the average crystal packing and therefore increase the likelihood of observable changes in the lattice parameters. Follow-up solid-state simulations may provide additional insight into this. Higher-energy tautomers may also inhibit crystallization and/or generate defects,^37,38^ but since Form III is obtained as a crystalline powder, it is hard to directly assess these secondary effects.

By contrast, in aqueous solution, the triketo tautomer predominates by a wide margin. Even at the boiling point of water, the triketo tautomer is 99.5% abundant. It is therefore not surprising that solution growth methods consistently yield Form I composed of a singular triketo tautomer.

### Form I at High Temperature

The general shape of the Form III PXRD pattern bore a striking similarity to the high-temperature PXRD pattern of UA Form I previously reported by Izatulina et al.^26^ Using a sample obtained from a renal stone, they collected PXRD data at temperatures up to 380 °C. A distinct change in the PXRD pattern was observed at temperatures above 220 C° at which point “two diffraction maxima··· merge into one peak”.^26^ The 2θ peak positions in ref 26 reference values obtained with Co Kα. When converted to a standard Cu Kα scale, the merging peaks they refer to fall between 2θ = 27 and 28°.

In an effort to discern whether Form I converts to Form III at high temperature, we performed our own in situ synchrotron powder diffraction study. A commercial sample of UA Form I (99+% purity) was heated at 10 °C/min from 25 to 300 °C with continuous data acquisition. The use of sPXRD enables shorter exposure times and significantly faster data collection compared to conventional PXRD. Representative sPXRD patterns of Form I collected between 25 and 300 °C appear in blue in Figure 5. Our results qualitatively reproduce the previous report^26^ though the merging of (400) and (121) peaks between 2θ = 27 and 28° began at slightly lower temperatures. For expanded views of select reflections, see Figure S11.

**Figure 5:**
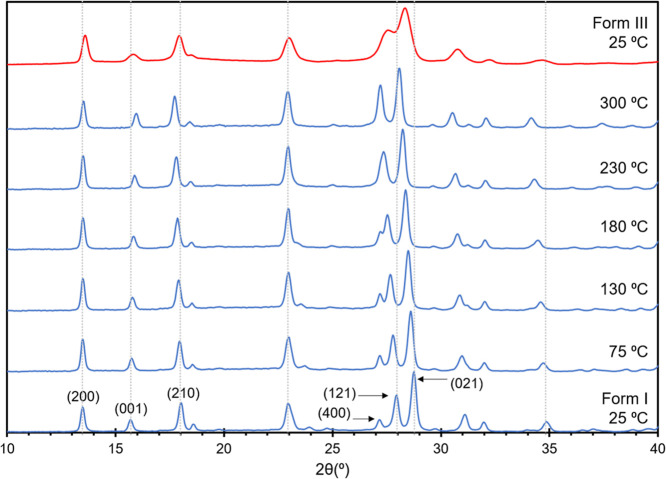
sPXRD patterns of UA Form I at varying temperatures (blue) and the room-temperature PXRD pattern of Form III (red). Vertical gray lines are included as a visual guide. Miller indices correspond to the cell convention reported in Table 1.

Thermal expansion in Form I occurs mainly along the *b*-axis (π stack), while the *c*-axis contracts. The *a*-axis does not appreciably change over the temperature range investigated. Whether intermolecular H transfer or tautomerization occurs in Form I at high temperature is an interesting question, but not one that can be adequately assessed based on the available data. We do know that when Form I is cooled from 300 °C back to 25 °C, it returns to the original room-temperature PXRD pattern. Thus, in the event H transfer does occur at higher temperatures, it would appear to be a reversible process. For the higher-energy tautomers in Form III, there appears to be no obvious route to relaxing to the triketo form other than via a dissolution–recrystallization mechanism. Direct comparison of the powder patterns for Form III at 25 °C (in red) and Form I at >200 °C confirms they are similar but not identical.

## Conclusions

This paper reports the discovery and characterization of the first new polymorph of anhydrous uric acid (Form III) in more than a half century. Unlike the well-known Form I, which crystallizes from aqueous solution, Form III is produced under high-temperature thermal decomposition conditions. While Forms I and III share similar topologies, they can be clearly distinguished by their powder X-ray diffraction patterns.^39^ Many possible interpretations of the unit cell differences between Form I and Form III were considered. Among these, the most plausible explanation is that the Form III crystal lattice accommodates a sizable number of molecules in tautomeric states other than the triketo minima. Support for this hypothesis comes from DFT calculations, which predict that a significant population of higher-energy tautomers may be present under the conditions used to synthesize Form III. Observed differences in the electron delocalization in Forms I and III are also consistent with this interpretation. Similarities in the PXRD patterns of high-temperature Form I and room-temperature Form III were carefully considered but ultimately led to the conclusion that the two are not identical. Overall, the work highlights how synthetic conditions—particularly temperature—can influence tautomer populations and enable access to previously unknown polymorphic forms.

## Supplementary Material



## Data Availability

Powder patterns for Forms I, II, and III were also deposited with the ICDD. The data files can be requested at pdj@icdd.com.

## Notes

PXRD patterns, FTIR spectra, Rietveld refinement data, electron density isosurface plots, and calculated tautomer energies (PDF)
