# Supplementary material for: A Uric Acid Polymorph Generated via Thermal Decomposition
Source: Cryst Growth Des. 2026 Jul 22;26(15):6098–106. doi: 10.1021/acs.cgd.6c00610 (PMC13449764; doi:10.1021/acs.cgd.6c00610)
Supplement: Supplementary file 1 [file cg6c00610_si_001.pdf]

Supplementary Information

A Uric Acid Polymorph Generated via Thermal Decomposition

Josephine Bicknell[1], Shae S. London[1], Talaidh L. Isaacs[1], Amanda K. Rutledge[1], Lindsey K. Foote[1], Alyssa M. Thornton[1], Timothy G. Fawcett[2], and Jennifer A. Swift*[1]

[1] Department of Chemistry, Georgetown University, Washington, DC 20057 USA

[2] International Centre for Diffraction Data, Newtown Square, PA 19073 USA

Table of Contents

page

Figure S1. Offset view of experimental PXRD patterns of uric acid Forms I (blue), II (gray) and III (red) shown in Figure 1. Form I is commercial material, Form II is dehydrated UAD, and Form III is decomposed AUH. Miller Indices correspond to Form I (URICAC). 3

Figure S2. PXRD pattern of Form I uric acid. The experimental pattern of commercial uric acid powder (blue) and the simulated PXRD from the single crystal URICAC structure (black) are shown. 4

Figure S3. PXRD pattern of Form II uric acid. The experimental pattern of dehydrated UAD (green) and the PXRD pattern reported by Shirley and Sutor (black) are shown. 4

Figure S4. Synthetic ammonium urate hydrate heated for different times and temperatures. (A) From bottom: (turquoise) unheated, and after heating for 1 hour at (pink) 150 °C, (blue) 190 °C, (orange) 220 °C and (green) 260 °C. The 260 °C samples shows partial decomposition to UA Form III. (B) From bottom: (red) unheated, (blue) air dried at room temperature for 4 days, and after heating (green) 150 °C for 2 days, (pink) 170 °C for 2 days, and (turquoise) 200 °C for 3 days. The 200 °C (3 days) sample decomposed to UA Form III. 5

Figure S5. PXRD of AUH and UA Form III. (from bottom): AUH synthesized from UA Form I starting material. Heating AUH to 400 °C generates UA Form III. Immersion of UA Form III in 25% ammonium hydroxide solution regenerates AUH. Heating the regenerated AUH to 400 °C again yields UA Form III. 6

Figure S6. FTIR spectra of uric acid Forms. Form I is commercial uric acid powder, Form II was prepared by heating UAD to 200°C, Form III was prepared heating AUH to 400°C. Spectra are offset along the y-axis for clarity. For closeups of the region between 1600 – 700 cm^{-1}, see Figure S6. 7

Figure S7. Closeups of the FTIR fingerprint region of Forms I, II and III with labeled peak positions. 8

Table S1. Multiple refinements for Uric Acid Form III prepared by decomposition of AUH. 9

Figure S8. Rietveld refinement of Form III uric acid. The difference plot is shown at the top. The black line is the experimental data, the refined pattern is shown in pink. The experimental pattern is from a sample of AUH decomposed by heating at 200°C for 3 days. 9

Figure S9. PXRD pattern of Form III uric acid. The experimental pattern (red) is from a sample of AUH decomposed by heating to 400°C. The simulated PXRD (black) is the best structure determined by Rietveld refinement. 10

Figure S10. Isosurfaces of Forms I and III at three different % rendering illustrate differences in the delocalization of electron density. At 50% rendering, this is most apparent between the bridgehead atoms. At 34% rendering, the connectivity across all O, N and C atoms is apparent only in Form I. For full connectivity to become apparent in Form III, rendering at 18% is necessary. 11

Table S2. Tautomer energies calculated at the M06-2X/cc-pVQZ level of theory compared against previously reported values. All energy values are reported in kcal/mol. 12

Table S3. Boltzman population of tautomers at different temperatures calculated from the single point energies. 12

Figure S11. (top) Expanded views of sPXRD peaks of Form I at variable temperature. Between 25 and 300 °C, the *b*-axis expands and the *c*-axis contracts. Dashed lines are included as a visual guide. Miller indices correspond to the cell convention reported in Table 1. (bottom) Pawley refined cell parameters for Form I at different temperatures. 13

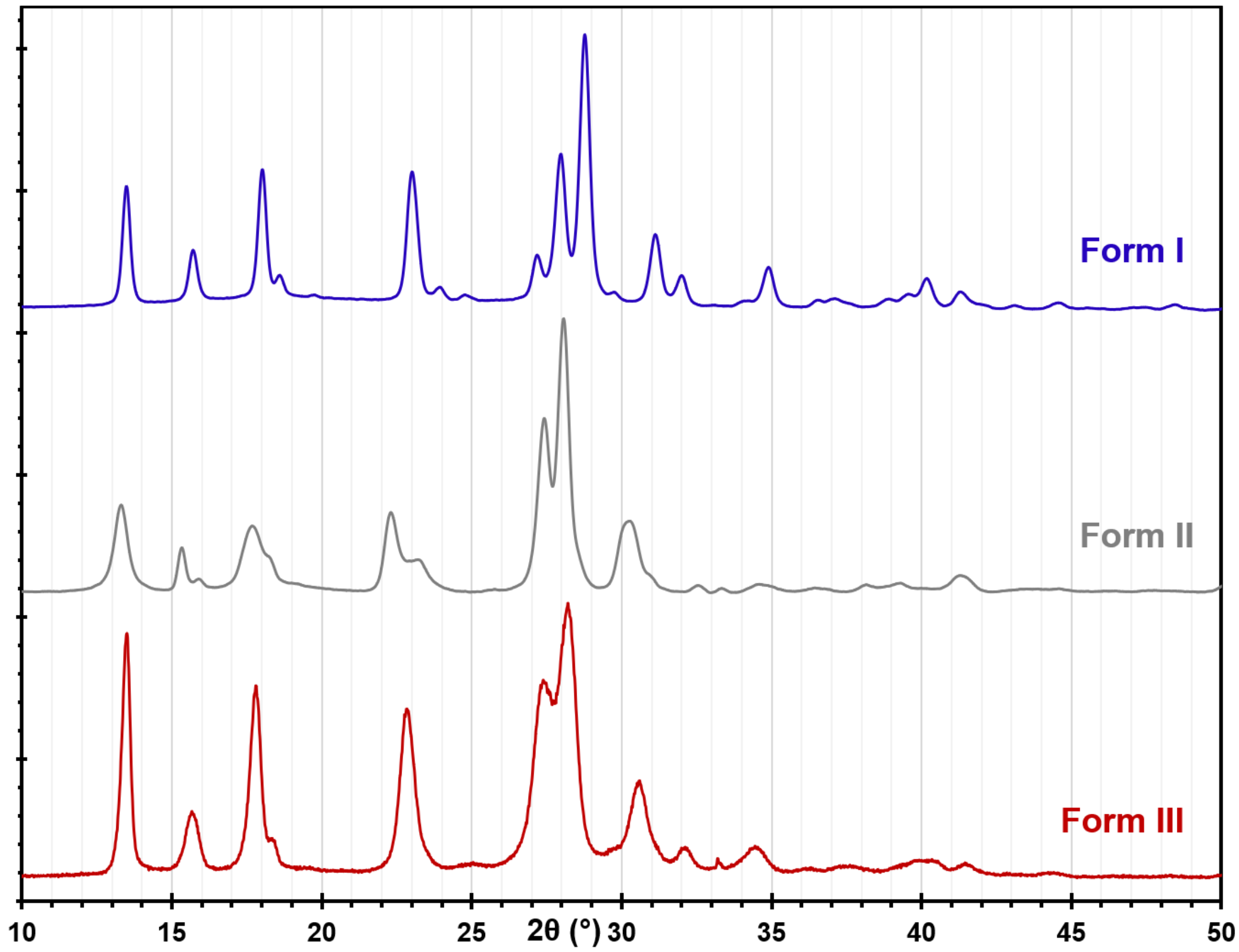

Figure S1. Offset view of experimental PXRD patterns of uric acid Forms I (blue), II (gray) and III (red) shown in Figure 1. Form I is commercial material, Form II is dehydrated UAD, and Form III is decomposed AUH. Miller Indices correspond to Form I (URICAC).

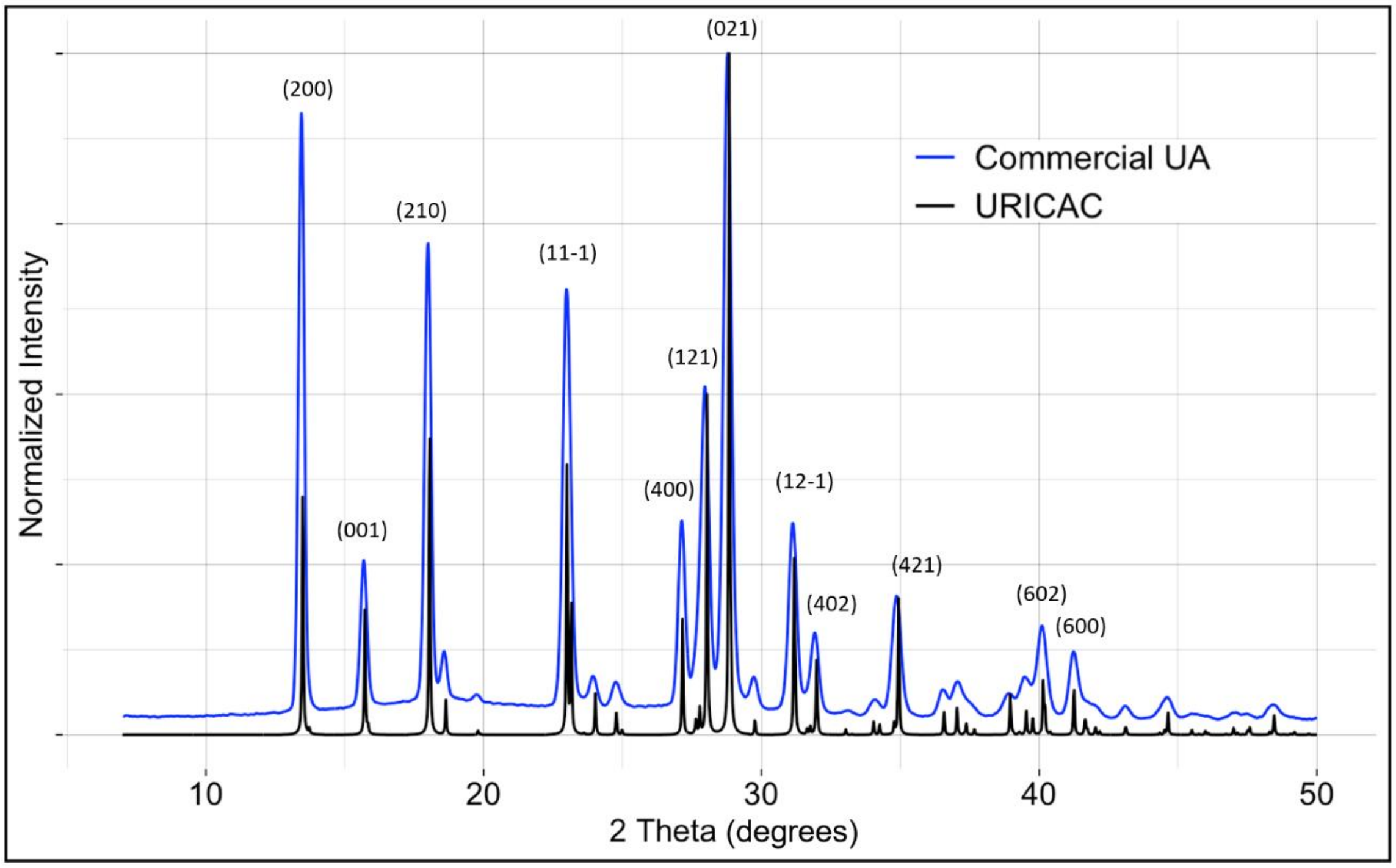

Figure S2. PXRD pattern of Form I uric acid. The experimental pattern of commercial uric acid powder (blue) and the simulated PXRD from the single crystal URICAC structure (black) are shown.

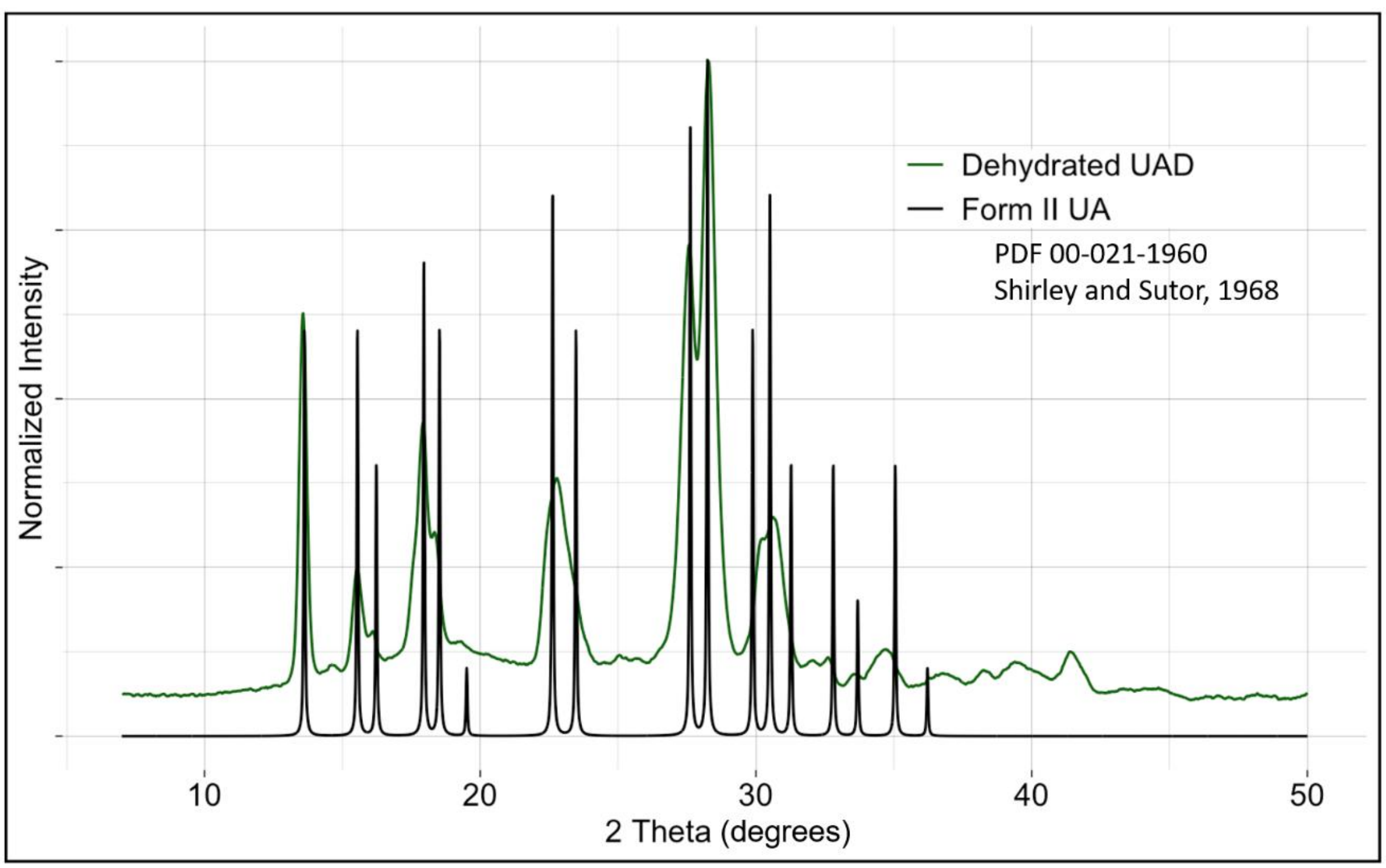

Figure S3. PXRD pattern of Form II uric acid. The experimental pattern of dehydrated UAD (green) and the PXRD pattern reported by Shirley and Sutor (black) are shown.

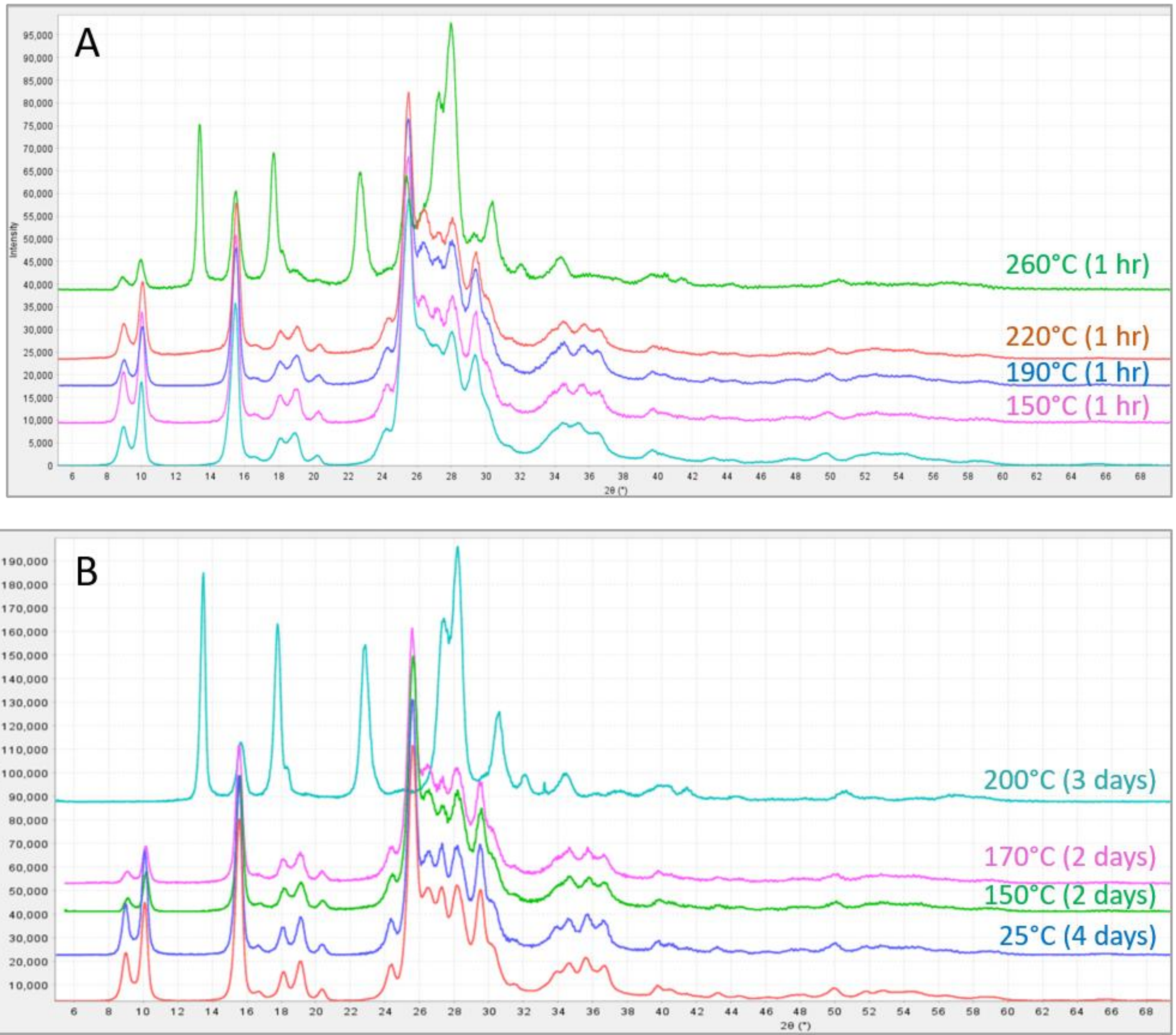

Figure S4. Synthetic ammonium urate hydrate heated for different times and temperatures. (A) From bottom: (turquoise) unheated, and after heating for 1 hour at (pink) 150 °C, (blue) 190 °C, (orange) 220 °C and (green) 260 °C. The 260 °C samples shows partial decomposition to UA Form III. (B) From bottom: (red) unheated, (blue) air dried at room temperature for 4 days, and after heating (green) 150 °C for 2 days, (pink) 170 °C for 2 days, and (turquoise) 200 °C for 3 days. The 200 °C (3 days) sample decomposed to UA Form III.

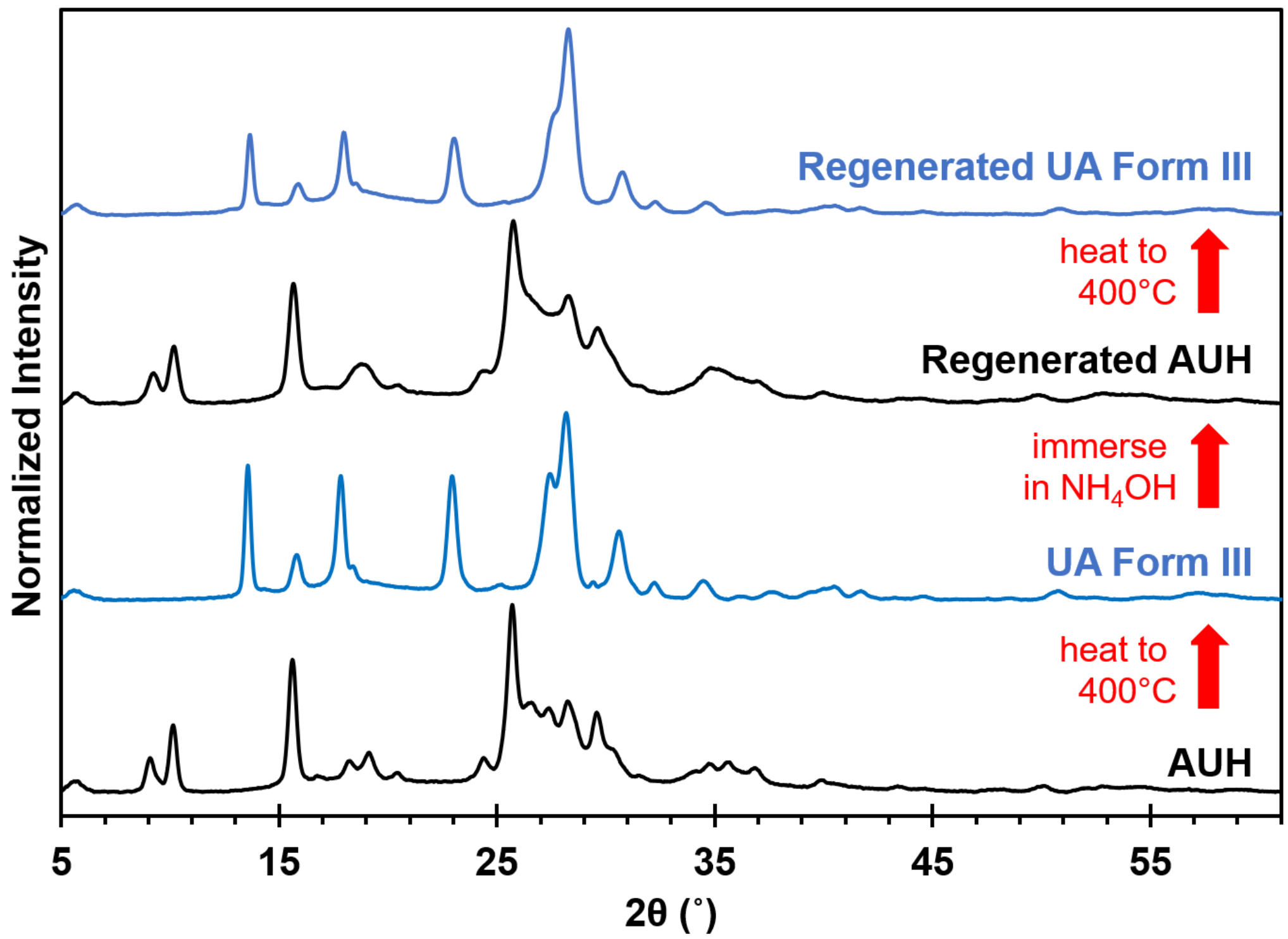

Figure S5. PXRD of AUH and UA Form III. (from bottom): AUH synthesized from UA Form I starting material. Heating AUH to 400 °C generates UA Form III. Immersion of UA Form III in 25% ammonium hydroxide solution regenerates AUH. Heating the regenerated AUH to 400 °C again yields UA Form III.

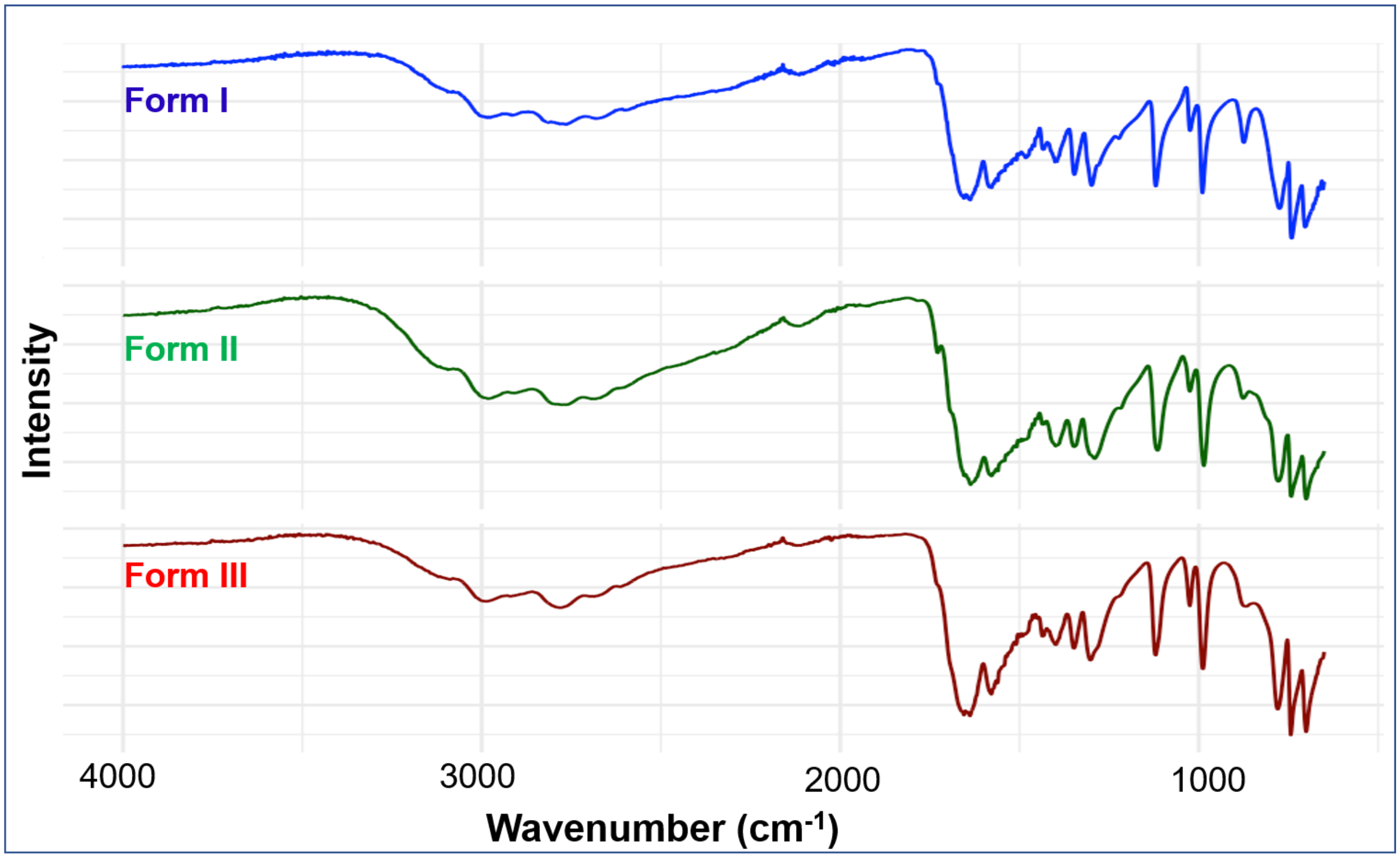

Figure S6. FTIR spectra of uric acid Forms. Form I is commercial uric acid powder, Form II was prepared by heating UAD to 200°C, Form III was prepared heating AUH to 400°C. Spectra are offset along the y-axis for clarity. For closeups of the region between 1600 – 700 cm^{-1}, see Figure S6.

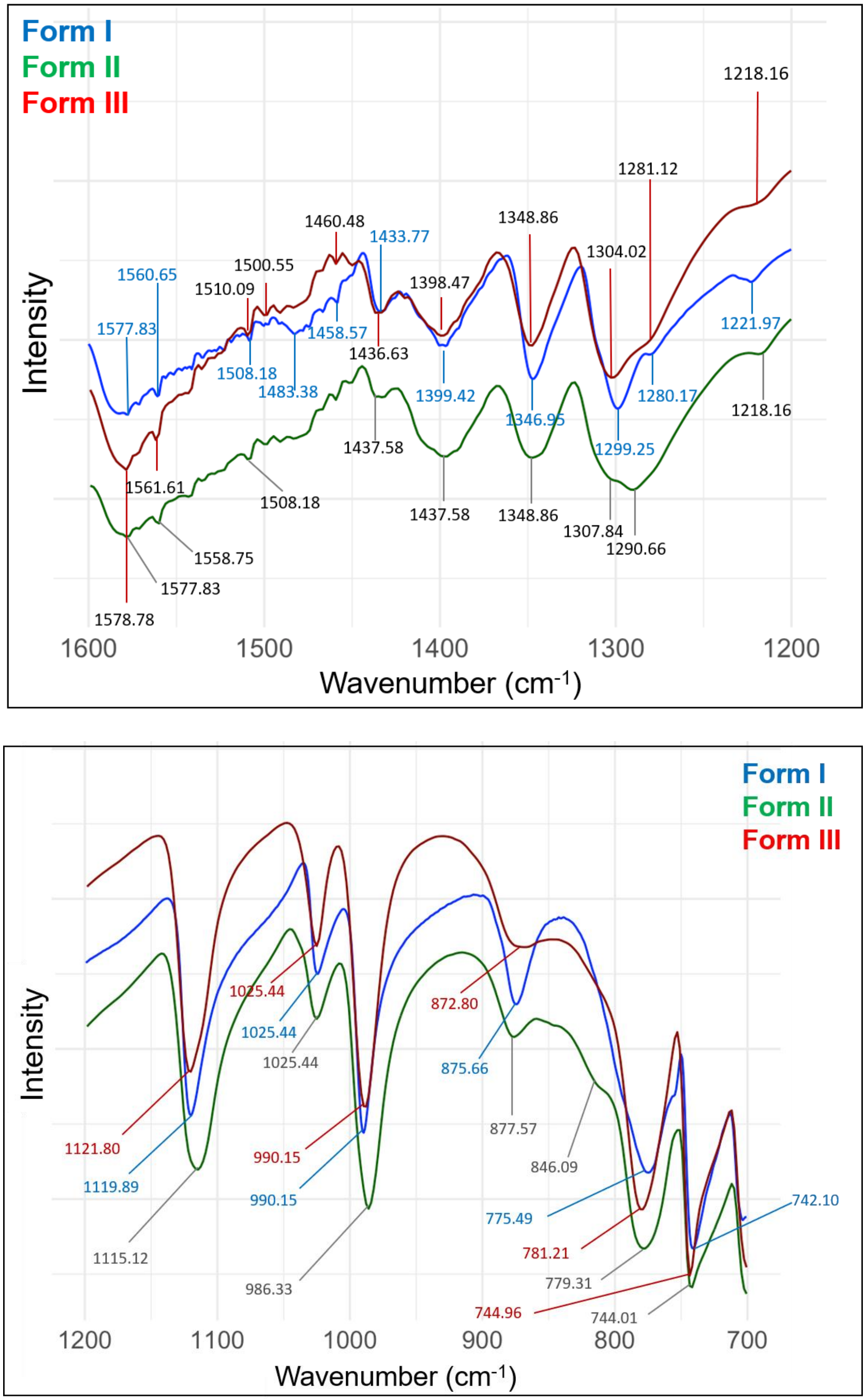

Figure S7. Closeups of the FTIR fingerprint region of Forms I, II and III with labeled peak positions.

Table S1. Multiple refinements for Uric Acid Form III prepared by decomposition of AUH.

Decomposition conditions	Heated 2°C/min to 400°C	Heated 2°C/min to 400°C	Heated 200°C for 4 days	Heated 200°C for 3 days
				BEST
	$P2_1/a$	$P2_1/a$	$P2_1/a$	$P2_1/a$
a (Å)	14.2856	14.3079	14.3419	14.30957
b (Å)	7.65104	7.62627	7.62587	7.62384
c (Å)	6.1419	6.16232	6.16963	6.16678
α (°)	90	90	90	90
β (°)	65.045°	65.1335°	65.2184°	65.12737
γ (°)	90	90	90	90
Volume (Å)	608.63	610.07	612.63	610.36
R_{wp}	5.5	4.78	6.42	5.13

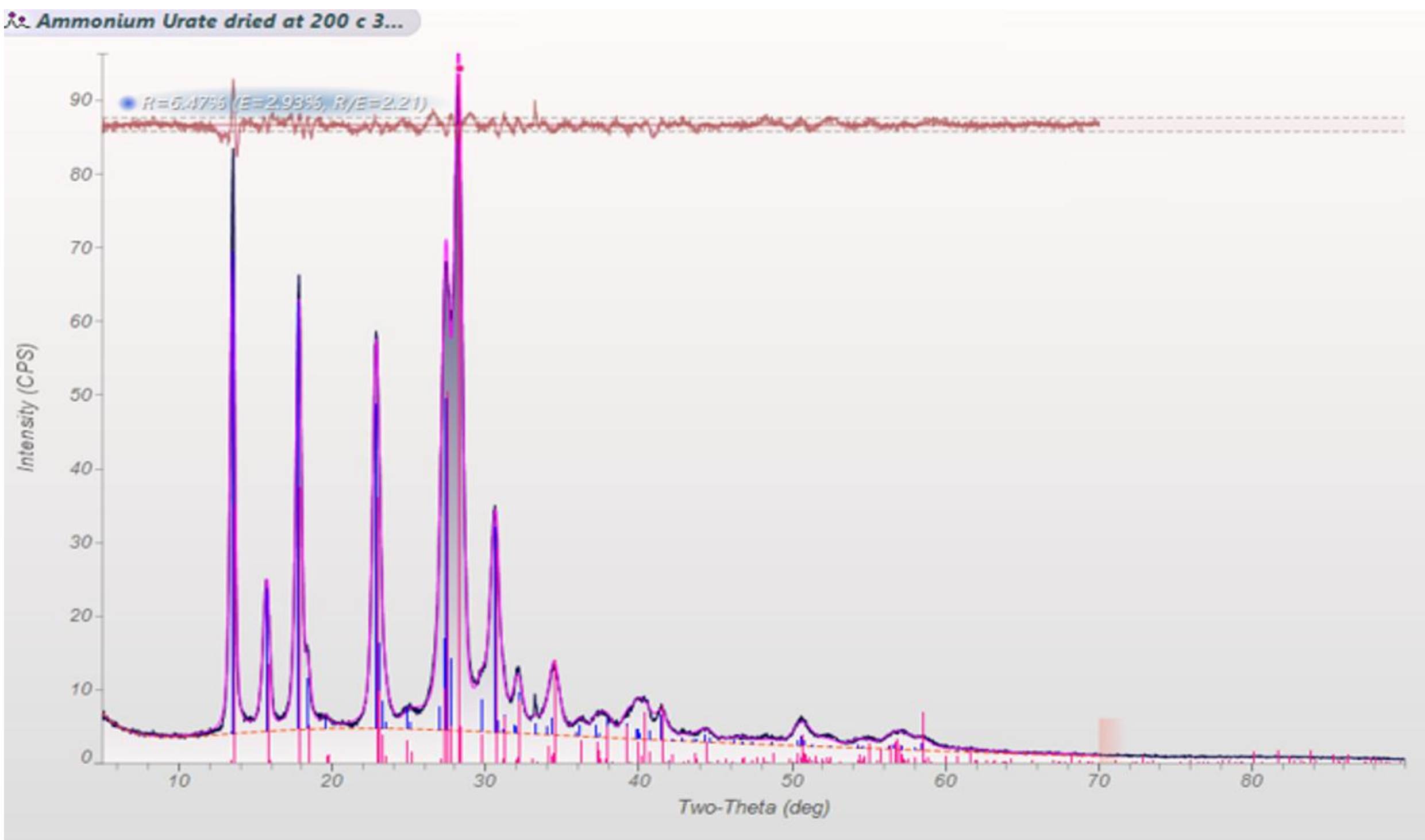

Figure S8. Rietveld refinement of Form III uric acid. The difference plot is shown at the top. The black line is the experimental data, the refined pattern is shown in pink. The experimental pattern is from a sample of AUH decomposed by heating at 200°C for 3 days.

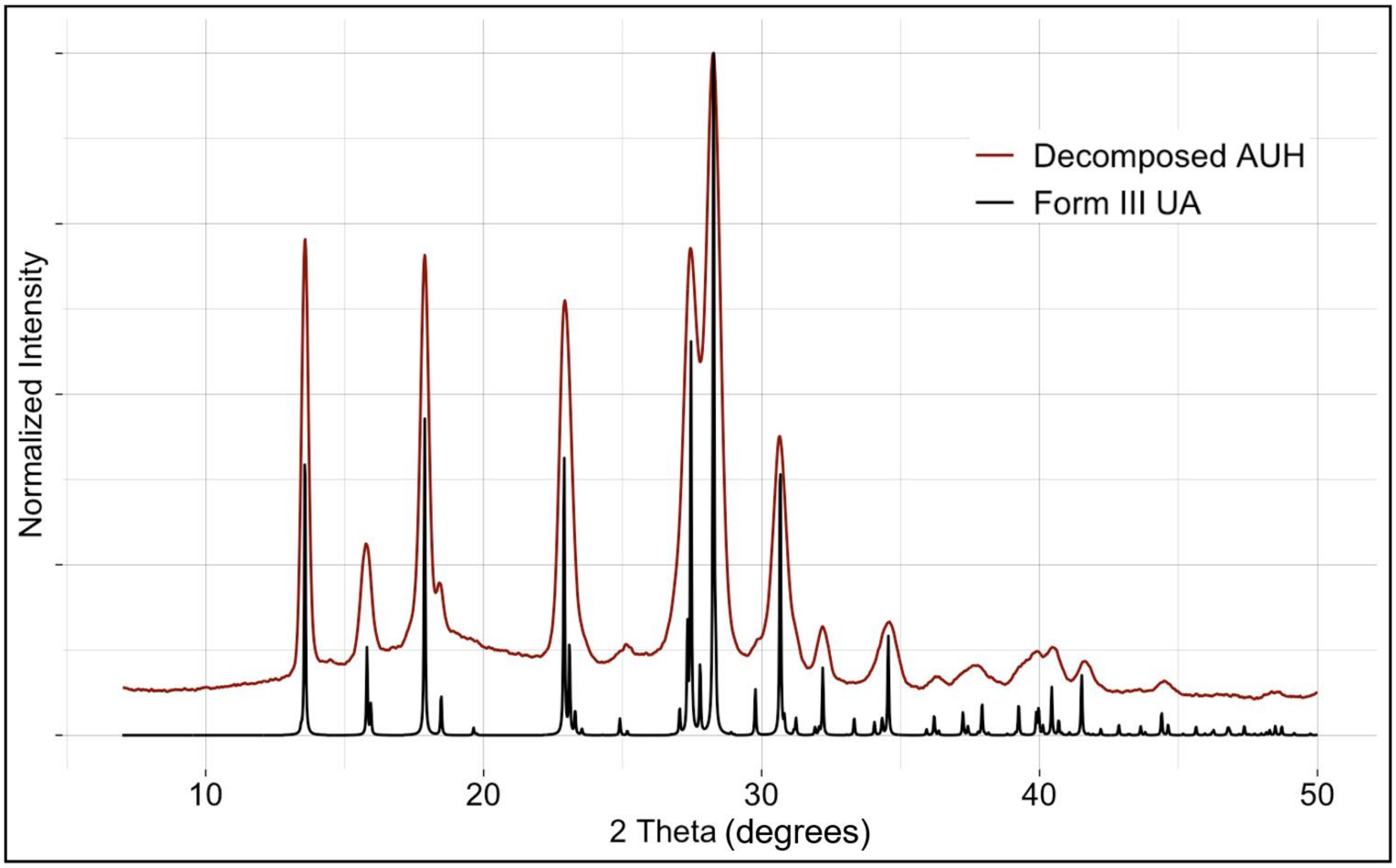

Figure S9. PXRD pattern of Form III uric acid. The experimental pattern (red) is from a sample of AUH decomposed by heating to 400°C. The simulated PXRD (black) is the best structure determined by Rietveld refinement.

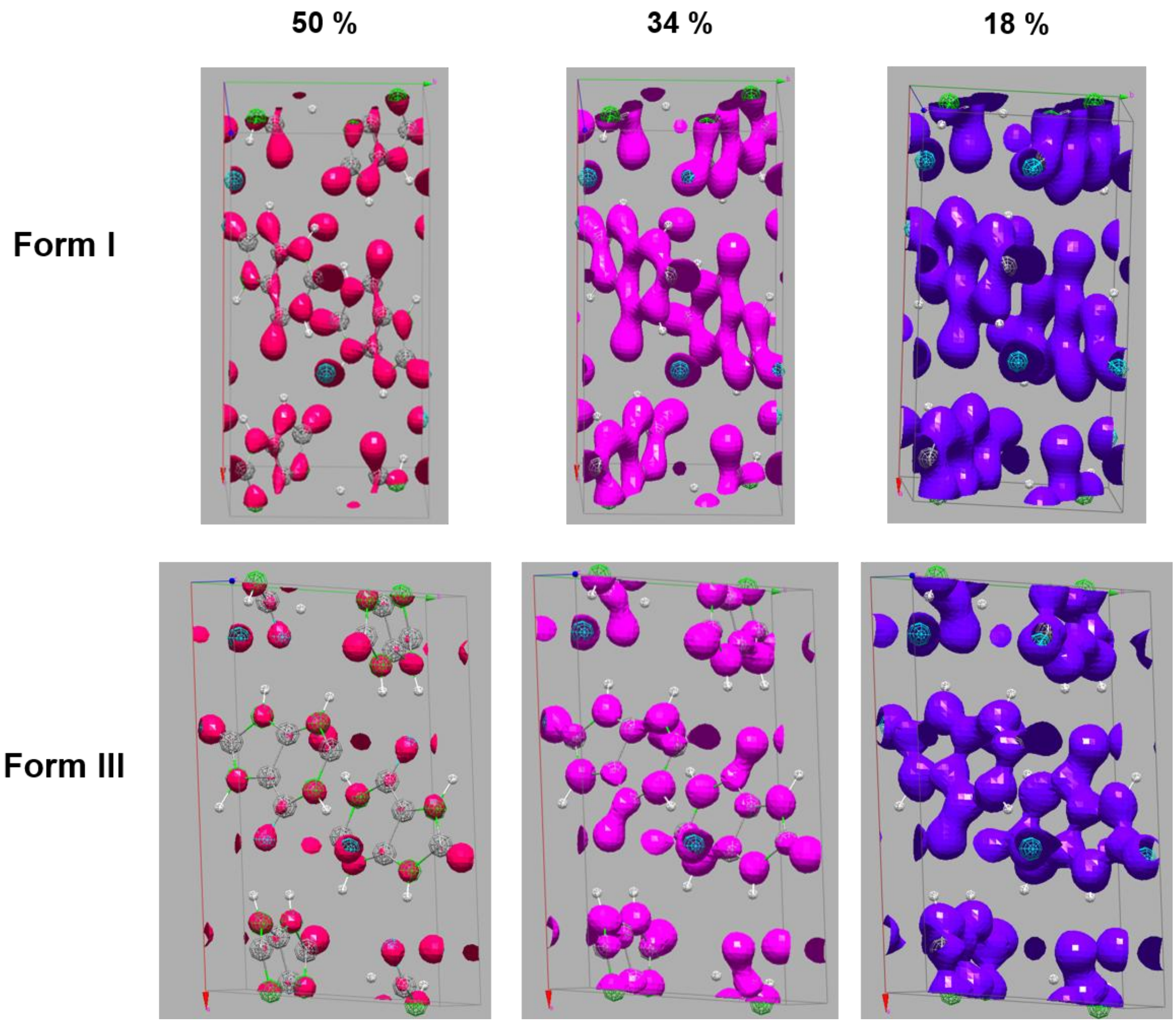

Figure S10. Isosurfaces of Forms I and III at three different % rendering illustrate differences in the delocalization of electron density. At 50% rendering, this is most apparent between the bridgehead atoms. At 34% rendering, the connectivity across all O, N and C atoms is apparent only in Form I. For full connectivity to become apparent in Form III, rendering at 18% is necessary.

Table S2. Tautomer energies calculated at the M06-2X/cc-pVQZ level of theory compared against previously reported values. All energy values are reported in kcal/mol.

	(M06-2X opt) M06-2X/cc-pVQZ		Lit.[a] AM1	Lit.[b] B3LYP/ 6-311++G(d,p)		Lit.[b] MP2/B3LYP	
	Gas	Aqueous	Gas	Gas	Aqueous	Gas	Aqueous
UA	0	0	0	0	0	0	0
N2_O1	1.71	4.14	6.8	3.7	5.5	1.6	3.1
N2_C3	9.65	10.14	7.6	-	-	-	-
N2_O2	15.26	8.65	11.1	-	-	-	-
N4_O3	1.61	5.03	5.9	4.9	5.5	1.5	5.2
N4_C3	10.65	10.32	9.2	-	-	-	-
N4_O2	17.14	11.21	17.3	-	-	-	-

(a) Raczyńska, E. D.; Makowski, M.; Szeląg, M.; Kamińska, B.; Zientara, K., *J. Molecular Structure: THEOCHEM* **2010**, *947*, 83-91, (b) Allen, R. N.; Shukla, M. K.; Leszczynski, J., *Int. J. Quantum Chem.* **2004**, *100*, 801-809.

Table S3. Boltzman population of tautomers at different temperatures calculated from the single point energies.

Gas Phase (M06-2X/cc-pVQZ)

Tautomer	(kcal/mol)	(kJ/mol)	25°C	100°C	200°C	300°C	400°C
URICAC	0.000	0.000	89.103	82.349	74.430	68.153	63.264
N2_C3	9.646	40.358	0.000	0.000	0.003	0.014	0.047
N2_O1	1.709	7.152	4.977	8.214	12.084	15.195	17.628
N2_O2	15.259	63.845	0.000	0.000	0.000	0.000	0.001
N4_C3	10.651	44.564	0.000	0.000	0.001	0.006	0.022
N4_O2	17.146	71.742	0.000	0.000	0.000	0.000	0.000
N4_O3	1.606	6.721	5.921	9.437	13.482	16.632	19.038

Aqueous Phase (M06-2X/cc-pVQZ, SMD model)

Tautomer	(kcal/mol)	(kJ/mol)	25°C	100°C	200°C	300°C	400°C
URICAC	0.000	0.000	99.887	99.511	98.312	96.215	93.336
N2_C3	10.137	42.413	0.000	0.000	0.002	0.013	0.048
N2_O1	4.138	17.315	0.092	0.375	1.205	2.542	4.231
N2_O2	8.651	36.196	0.000	0.001	0.010	0.048	0.145
N4_C3	10.321	43.184	0.000	0.000	0.002	0.011	0.042
N4_O2	11.206	46.886	0.000	0.000	0.001	0.005	0.021
N4_O3	5.027	21.033	0.021	0.113	0.468	1.165	2.177

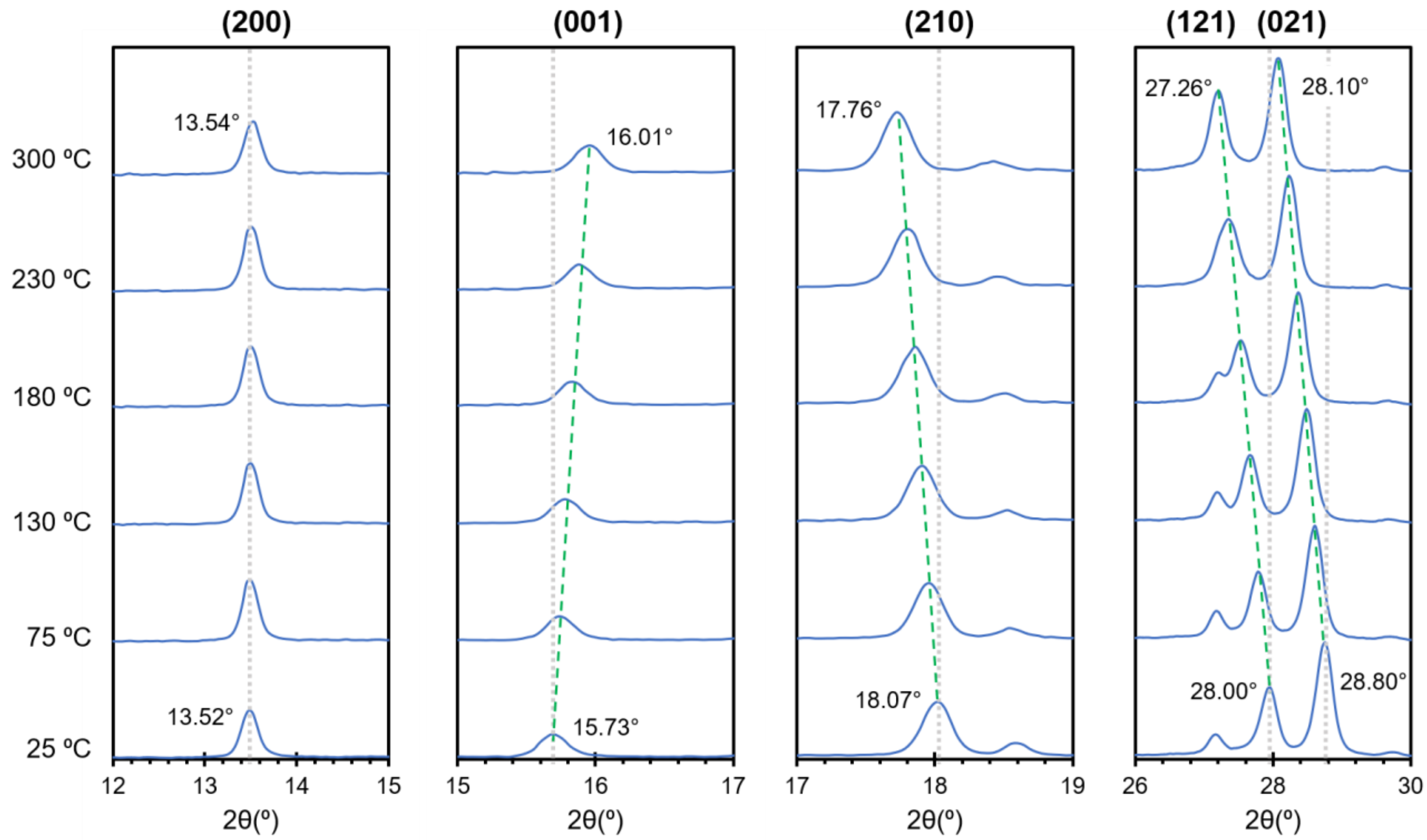

T (ºC)	a (Å)	b (Å)	c (Å)	β (°)	Volume (Å³)
25	14.440 (0.005)	7.4273 (0.005)	6.2068 (0.005)	65.18 (0.01)	604.20
75	14.407 (0.010)	7.4634 (0.011)	6.1890 (0.010)	65.04 (0.03)	603.31
130	14.433 (0.008)	7.5330 (0.009)	6.1792 (0.008)	65.02 (0.02)	608.95
180	14.461 (0.008)	7.6003 (0.008)	6.1702 (0.008)	64.91 (0.02)	614.12
230	14.425 (0.010)	7.6412 (0.011)	6.1395 (0.010)	64.67 (0.03)	611.68

Figure S11. (top) Expanded views of sPXRD peaks of Form I at variable temperature. Between 25 and 300 °C, the *b*-axis expands and the *c*-axis contracts. Dashed lines are included as a visual guide. Miller indices correspond to the cell convention reported in Table 1. (bottom) Pawley refined cell parameters for Form I at different temperatures.
